# VesselRegNet: Multi-Scale Feature Fusion Network for Multi-Modal Joint Retinal Vessel Segmentation and Registration

**DOI:** 10.3390/bioengineering13101179

**Published:** 2026-10-09

**Authors:** Anyu Cao, Maosong Jiang, Qinghong Gao, Xuelian Yang, Caiye Fan, Shu’an Liu, Zuoping Tan, Yuanyuan Wang

**Affiliations:** 1 School of Ophthalmology & Optometry, Wenzhou Medical University, Wenzhou 325000, China; 2 School of Data Science and Artificial Intelligence, Wenzhou University of Technology, Wenzhou 325000, China

**Keywords:** multimodal retinal image registration, multi-scale feature fusion, joint segmentation and registration, semantic registration, deformable registration

## Abstract

Multimodal retinal image registration remains challenging due to nonlinear intensity discrepancies and insufficient feature representation across modalities. To address these limitations, we propose VesselRegNet, a joint retinal vessel segmentation and registration framework based on multi-scale feature fusion. The model integrates a multi-scale semantic feature extraction module with a shared prediction head and rotation-equivariant constraints to enhance feature consistency. Unlike conventional approaches relying on binary segmentation masks, the proposed method utilizes dense semantic features to directly guide deformable registration. Experimental results demonstrate that VesselRegNet achieves an average Dice coefficient of 0.664 and SSIM of 0.645, achieving a relative improvement of 4.1% in Dice and 2.5% in SSIM over the strongest baseline (RetinaRegNet), and up to 11.9% improvement over general unsupervised registration frameworks. The proposed multi-scale fusion and joint learning strategy improves registration accuracy under complex retinal deformations.

## 1. Introduction

Multimodal retinal image registration is critical for clinical diagnosis. However, it remains challenging due to nonlinear intensity discrepancies, structural deformations, and limited anatomical overlap between modalities such as CFP and FFA. Existing methods, including traditional feature-based approaches and deep-learning frameworks, often suffer from insufficient multi-scale representation and sensitivity to rotational and pathological variations, leading to suboptimal performance [1,2].

**Figure 1:**
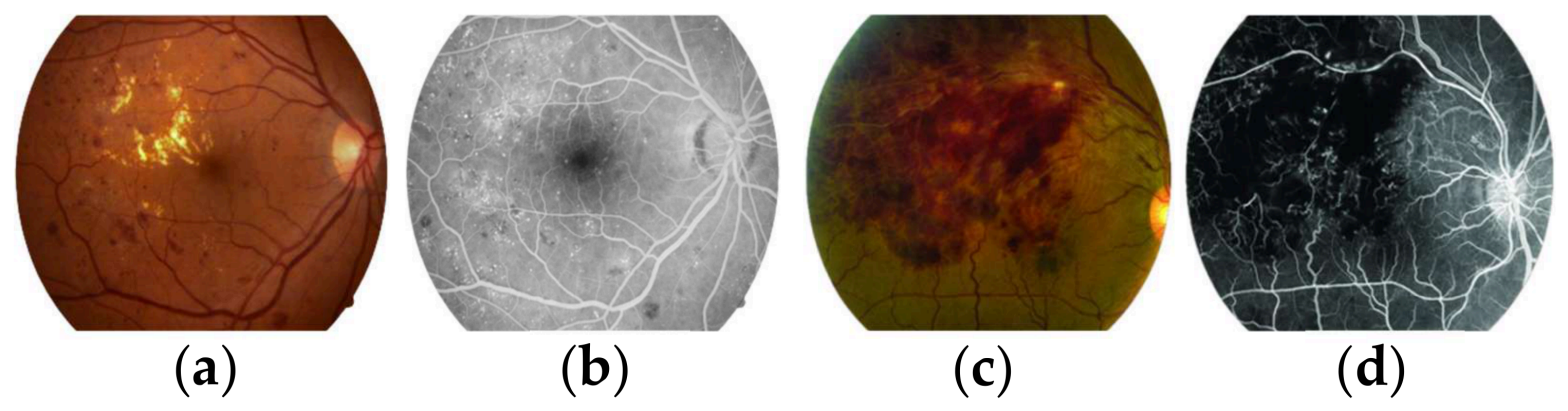
Comparison of color fundus photography (CFP) and fluorescein angiography (FFA) modalities: (**a**) (DR-CFP), (**b**) (DR-FFA), (**c**) (RVO-CFP), and (**d**) (RVO-FFA).

As shown in Figure 1, multimodal retinal images provide complementary clinical information. In diabetic retinopathy (DR), color fundus images reveal microaneurysms, hemorrhagic spots, and hard exudates, whereas fluorescein angiography highlights vascular leakage and hyperfluorescent regions. Similarly, in retinal vein occlusion (RVO), CFP images capture structural abnormalities, while FFA images reveal perfusion delays and non-perfused regions [3]. These differences demonstrate the importance of multimodal registration for comprehensive analysis.

This complementarity of multimodal retinal imaging—particularly in vascular structures—underlines the clinical value of accurate registration. However, differences in imaging principles between sensors often lead to low contrast, nonlinear intensity variations, and insufficient anatomical overlap [1], which significantly complicates precise image alignment.

For example, nonlinear discrepancies exist between the natural color contrast of color fundus photography (CFP) and the fluorescence intensity gradient of fluorescein angiography (FFA) [4]. In addition, images acquired in clinical settings are frequently degraded by pathological changes, such as hemorrhage occlusion in diabetic retinopathy (DR) or blurred boundaries in non-perfused areas of retinal vein occlusion (RVO) [5]. These factors hinder the reliable extraction of mutual information across modalities [6], thereby posing significant challenges to accurate retinal image registration.

Traditional retinal image registration methods rely primarily on intensity similarity or manual feature extraction—such as rigid registration based on the scale-invariant feature transform (SIFT) [7] or speeded-up robust features (SURF) algorithms [8]—and non-rigid deformation models optimized by mutual information [9]. Although effective in single-modality scenarios, these methods can be sensitive to nonlinear intensity variations across modalities, resulting in limited accuracy [1]. In recent years, deep-learning frameworks have advanced significantly in this field. Unsupervised registration networks such as the VoxelMorph [10] and TransMorph [11] frameworks use convolutional neural networks (CNNs) to predict deformation fields, enabling end-to-end optimization. However, they still face challenges such as insufficient multi-scale feature fusion and sensitivity to rotational deformation. While demonstrating potential on public datasets, these methods exhibit reduced registration accuracy with multimodal data (for example, FFA-CFP pairs), struggle to effectively handle intensity nonlinearity differences, and fail to address pathology-induced deformation and occlusions [2]. Consequently, more refined multi-scale mechanisms are required to address the complexity of retinal pathologies.

To overcome these limitations, this study proposes VesselRegNet, a joint vessel segmentation and deformable registration framework based on multi-scale feature fusion [12]. By integrating multi-scale semantic feature fusion, rotation-equivariant constraints [13], and semantically driven registration with end-to-end joint optimization, the proposed method aims to improve alignment accuracy in the presence of complex clinical deformations.

## 2. Related Work

### 2.1. Traditional Methods

Early registration methods for achieving the precise registration of retinal multimodal images can be broadly categorized into two types—that is, intensity- and feature-based methods [1]. Intensity-based methods treat registration as an optimization problem by employing similarity measures such as mutual information (MI) [14] or cross-correlation (CC) [15] for iterative registration. However, this approach can be sensitive to variations in lighting, differences in contrast, and shifts caused by retinal pathologies [16]. By contrast, feature-based methods demonstrate superior performance in retinal image registration [17]. This approach involves extracting keypoints, describing keypoints, matching corresponding keypoints, and estimating geometric transformations.

**Figure 2:**
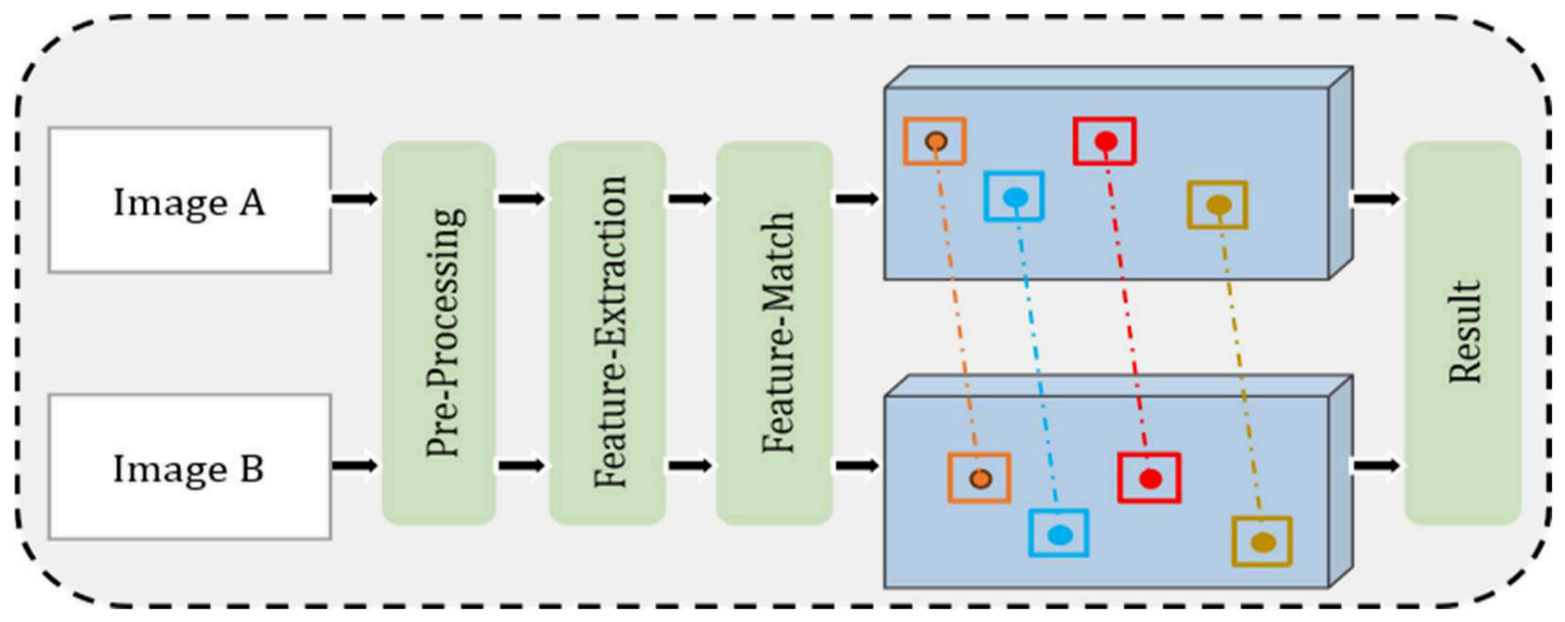
Feature-based registration flowchart.

The registration workflow, as illustrated in Figure 2, begins with the input of Moving image A and fixed image B, after which the images are preprocessed to extract their features. During the feature-matching phase, the extracted features are aligned, yielding the final output.

Feature-based methods can extract prominent retinal landmarks, including vascular bifurcations, vessel centerlines, or the optic disc for matching [18]. The dual-bootstrap iterative closest point (ICP) algorithm [19] can be used to align images by iteratively refining the vascular landmark matching. Other methods include tree matching for temporal registration [20], landmark matching combined with linear programming [21], or using the generalized dual-bootstrap ICP algorithm for multimodal registration [22]. Descriptors such as SIFT variants [7], partial intensity-invariant features [23,24], or direction-agnostic matching [13] have been employed to address multimodal differences. Although robust to certain deformations, these methods can be computationally expensive, sensitive to low-quality images, and limited in their handling of nonlinear intensity mapping across modalities [25].

### 2.2. Deep-Learning-Based Approach

**Figure 3:**
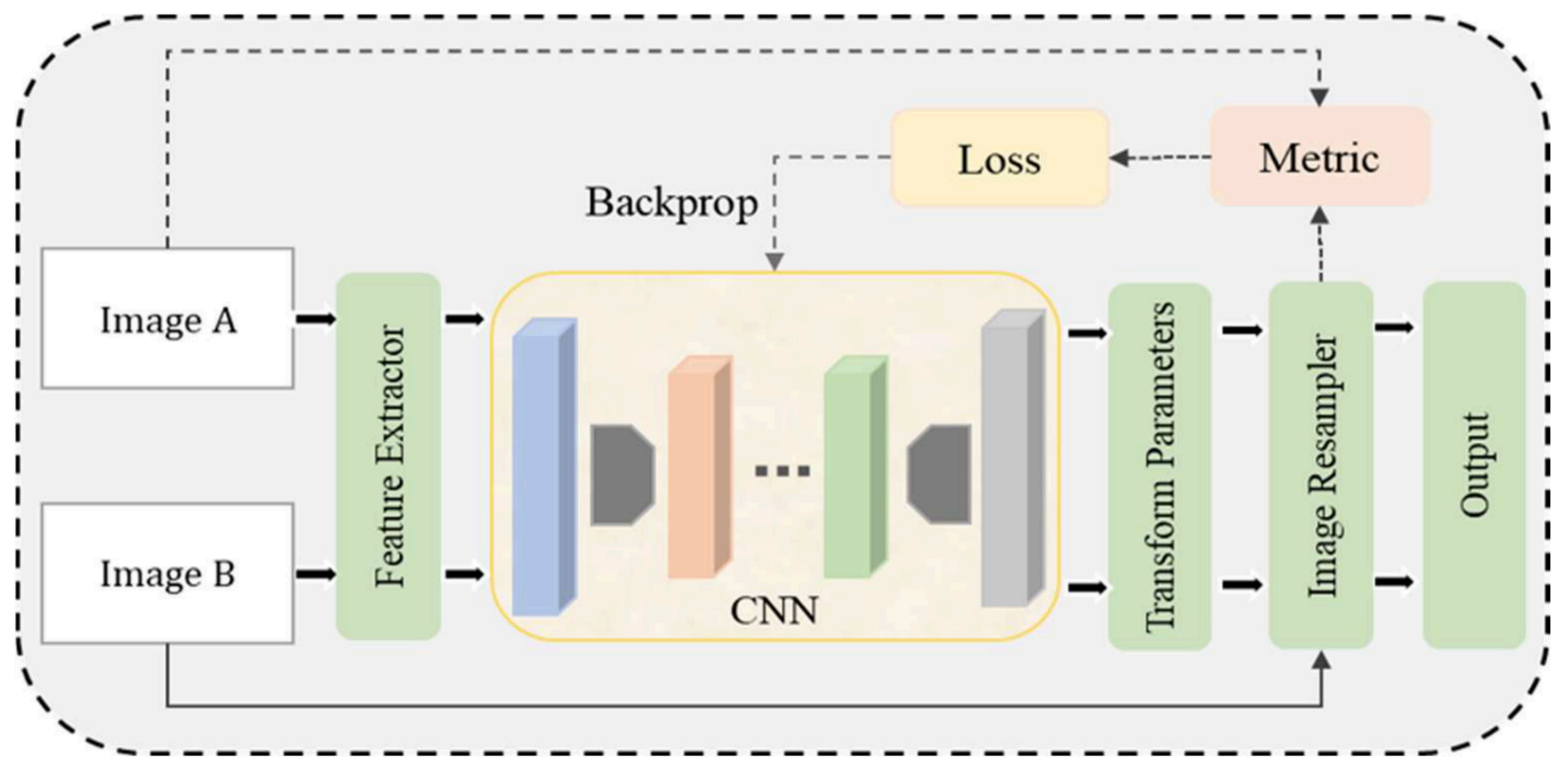
Deep learning-based registration workflow (unsupervised).

The deep-learning-based approach uses CNNs to learn image features automatically, thereby considerably enhancing the image registration accuracy and robustness [26]. The specific workflow is illustrated in Figure 3; here, moving image A and fixed image B are input into the network to generate deformation fields, enabling deformation interpolation for the moving image. During training, a loss function is computed using the deformed and fixed images, and backpropagation is iteratively applied to optimize the results, ultimately yielding the registered image.

Deep-learning-based methods have advanced registration research by estimating the deformation fields [27]. Typical unsupervised frameworks—such as the VoxelMorph framework—use CNNs to predict dense displacement fields [10] optimized through similarity loss and regularization terms, and their extended variants introduce differential isomorphisms to ensure topological preservation via static velocity fields. Transformer-based models address the shortcomings of the ConvNet algorithm in terms of capturing long-range dependencies [11]. For example, the TransMorph model employs a hybrid transformer-ConvNet architecture that applies Swin Transformers in the encoder for global attention, while using convolutions in the decoder for local refinement, achieving higher Dice scores in tasks such as brain magnetic resonance imaging (MRI) [28]. Although these models perform well in general medical imaging, adapting them for retinal applications requires addressing specific problems, such as the sparse vascular structures in retinal images [29]. Moreover, current methods are often constrained by linear transformations; consequently, the diversity of retinal registrations remains limited.

### 2.3. Joint Segmentation and Registration

Joint segmentation and registration methods can enhance multimodal image registration accuracy by integrating both tasks within a unified framework and leveraging the semantic information from segmentation to guide registration [30]. Early methods predominantly employed a separate workflow, extracting vessel centerlines or bifurcation points, followed by landmark matching [31]. However, errors in the segmentation outputs could propagate directly into the registration stage. With advances in deep learning, end-to-end joint frameworks have gained prominence. For example, the VoxelMorph-seg model incorporates segmentation-assisted loss into unsupervised registration networks, and constrains deformation fields via segmentation masks [10]. DeepAtlas jointly learns image registration and segmentation networks in a semi-supervised manner, simultaneously improving both tasks on brain and knee MRI [32].

However, most existing joint frameworks rely on binary segmentation masks as intermediate representations, which lack sufficient structural information and limit registration accuracy [33]. In addition, segmentation and registration are often loosely coupled, resulting in insufficient feature interaction and can fail to fully leverage the complementary nature of both tasks at the feature level [34]. These limitations highlight the need for a more tightly integrated framework that leverages dense semantic features for improved multimodal alignment.

## 3. Materials and Methods

### 3.1. VesselRegNet Framework

**Figure 4:**
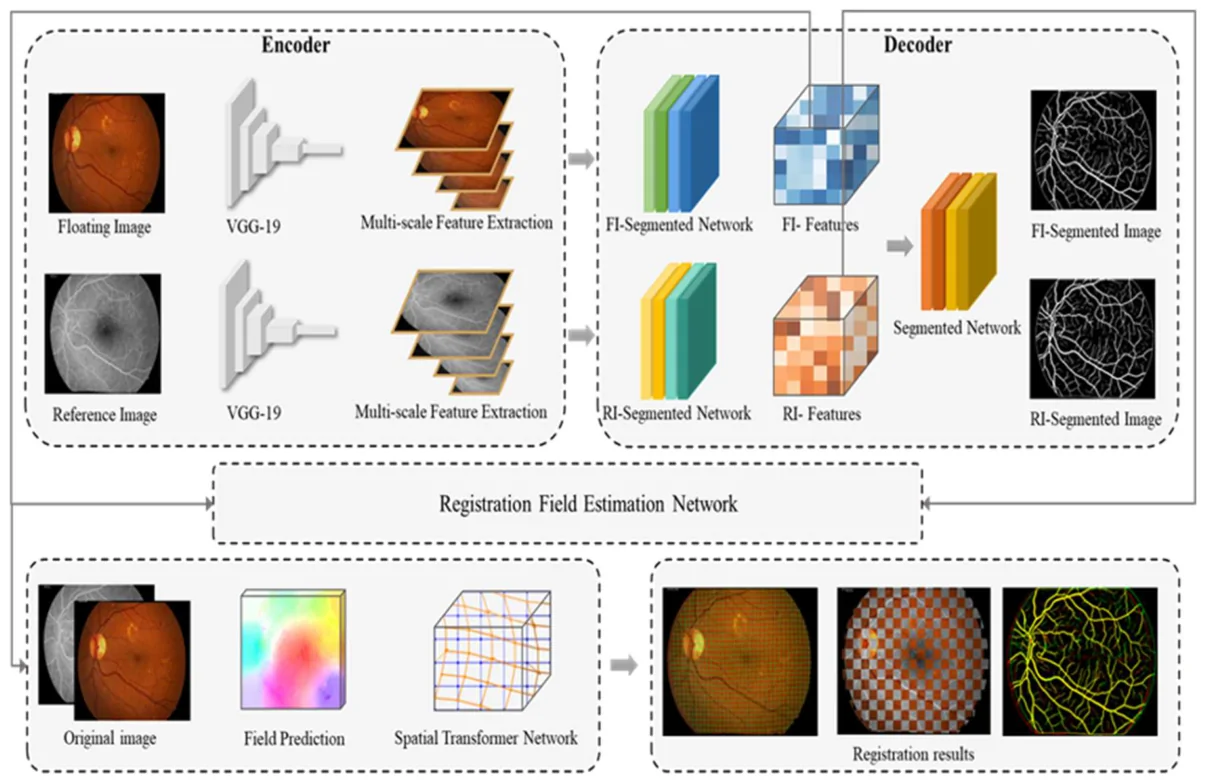
VesselRegNet registration framework.

The VesselRegNet framework is a joint retinal vascular segmentation and registration network based on multi-scale feature fusion that can achieve the semantic registration of multi-modal retinal images through a shared feature encoder. As shown in Figure 4, this framework comprises three core modules—that is, the multi-scale feature extraction module [35], joint segmentation module [36], and semantic registration module [37].

The framework employs a shared encoder to extract hierarchical multi-scale features from both the moving image (FFA) and fixed image (CFP). These features are fused into dense semantic representations, which are then utilized by a shared-prediction-head dual-branch segmentation network to generate vessel segmentation for both modalities. Subsequently, the dense semantic features from the two modalities are concatenated and fed into a semantic registration module to predict the dense deformation field. The entire process is trained end-to-end by jointly optimizing segmentation and registration objectives [38,39,40,41,42].

The detailed workflow of VesselRegNet is summarized in Algorithm 1.
**Algorithm 1**: VesselRegNet Joint Segmentation and Registration**Input**: Moving image $F_{A}$ (FFA), fixed image $F_{B}$ (CFP), style target mask $I_{style}$**Output:** Warped image $F_{A}^{\prime },$ segmentation maps $S_{A}^{\prime },$$S_{B}^{\prime }$ deformation field ϕ**Function VesselRegNet** ($F_{A},$$\;F_{B},$$\;I_{style}$)**:**
**1. Multi-scale extraction:** Compute$\left\{F_{A}^{l},\;\;F_{B}^{l}\right\}\;$(l = 1, …, 4) via pre-trained VGG-19
**2. Semantic fusion:** DRIU decoder upsamples/concatenates scales to yield 64-channel $G_{A},$$G_{B}$
**3. Joint segmentation:** Shared-head branches yield $S_{A}^{\prime }$= σ(Conv($G_{A};\theta_{s}$)), $S_{B}^{\prime }\;$= σ(Conv($G_{B};\theta_{s}$)); loss $L_{seg}$= $L_{style}$($S_{A}^{\prime },$$I_{style}$) + $L_{style}$($S_{B}^{\prime },$$I_{style}$) + $\lambda_{1}\cdot L_{rot}$
**4. Semantic registration:** Concat *G* = Concat($G_{A},$$\;G_{B}$); predict ϕ = UnetFlow(*G*); warp $F_{A}^{\prime }$ = STN($F_{A},$ϕ); losses $L_{reg}$= $\lambda_{2}\cdot L_{NCC}\;$($F_{A}^{\prime },$$\;F_{B}$) + $\lambda_{3}\cdot L_{smooth}+\lambda_{4}\cdot L_{ssim}\;$($F_{A}^{\prime },$$\;F_{B}$)
**5. Joint optimization:** Minimize $L_{total}$ = $L_{seg}$ + $L_{reg}(Equation(7))$ via backpropagation
**return**$\;F_{A}^{\prime },$$\;S_{A}^{\prime },$$\;S_{B}^{\prime },$ ϕend 

The overall workflow of the VesselRegNet framework is summarized in Algorithm 1. The network first extracts multi-scale features from both modalities and fuses them into dense semantic representations, which are subsequently used for joint vessel segmentation and semantically driven deformable registration through end-to-end optimization [43,44,45].

### 3.2. Multi-Scale Semantic Segmentation

The VesselRegNet segmentation module first employs a pre-trained VGG-19 encoder to extract multi-scale features ${\left\{F_{A}^{l},F_{B}^{l}\right\}}_{l=1}^{4}$ (channels 64/128/256/512) from the input FFA and CFP images, progressively capturing layers from texture details to higher-level semantic information. The DRIU decoder fusion mechanism is then used to generates 16-channel feature maps from each layer. These are unified to ¼-size resolution via deconvolution upsampling and bilinear interpolation, concatenated into a 64-channel semantic volume, where ⨁ denotes Concat and *k* traverses from shallow to deep layers. This fusion condenses the resolution differences, generating dense intermediate features ($G_{A}$ and $G_{B}$). This avoids binary-mask sparsity problems and is directly applied at the registration stage, defined as follows:

(1) $$G^{K}=\;\mathrm{Upsample}(F^{K})⨁G^{K+1},\;k=1,\;\ldots ,\;4$$

The shared prediction head dual-branch network performs regression from $G_{A}$ and $G_{B}$ to segment $S_{A}^{\prime }$ = $\sigma \left(Conv(G_{A};\theta_{S})\right)$, $S_{B}^{\prime }$ =$\;\sigma \left(Conv(G_{B};\theta_{S})\right)$, where σ denotes the sigmoid function and $\theta_{S}$ serves as a shared weight to ensure modal consistency.

Since the CF–FFA dataset provides no pixel-wise vessel annotations, pixel-wise supervision is not applicable. Following the weakly supervised style-transfer scheme, we supervise segmentation with a style loss: a single expert-annotated vessel map $I_{style}$ from the public HRF dataset [46] serves as the style target, and Gram matrices $G_{l}$ of frozen pre-trained VGG-19 features are used to match the global vessel statistics between the prediction and the style target:

(2) $$L_{style}=\;\sum_{l}{\left\|G_{l}(S_{x}^{\prime })-G_{l}\left(I_{style}\right)\right\|}_{F}^{2},x\in \left\{A,B\right\}$$

In addition, a rotation-equivariance self-consistency loss encourages orientation-independent vessel extraction by requiring the segmentation of a 180°-rotated input to match the rotated segmentation of the original input:

(3) $$L_{rot}=\sum_{x\in \left\{A,B\right\}}MSE({Rot}_{180}(Seg\left({Rot}_{180}\left(F_{x}\right)\right)),Seg(F_{x}))$$

**Figure 5:**
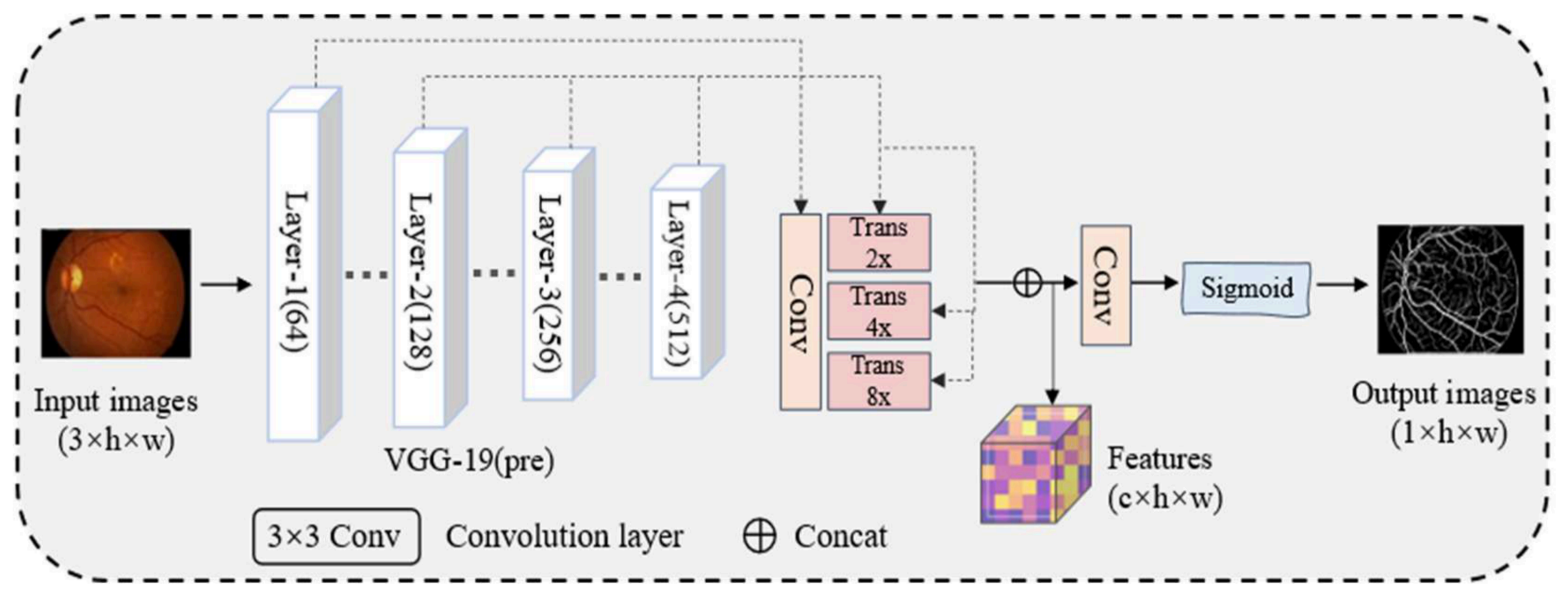
Architecture of the multi-scale semantic segmentation network.

Additionally, we employed an enhanced DRIU network for the vascular segmentation task, the architecture of which is illustrated in Figure 5. This network builds upon the pretrained VGG-19 model, retaining its fixed weights to extract low-level visual features (such as edge and line information) from the retinal images. New layers were added to progressively fuse these low-level features into higher-level representations of the vascular structures. To match the VGG-19’s input requirements, the single-channel segmentation map was expanded to a 3-channel image via channel duplication. Consequently, two segmentation networks with a shared architecture were constructed for the source (FFA) and target (CFP) images.

As shown in Figure 4 and Figure 5, the front-end sections of both networks operate independently, whereas the final layer (containing a sigmoid activation function) [47] constitutes a shared structure. During training, the 64-channel feature maps extracted from both independent components were passed to the subsequent registration steps to provide complementary retinal structural information. By sharing the final layer, the model could guide the input images from different modalities toward consistent feature representations, thereby enhancing the structural correspondence between modalities.

**Figure 6:**
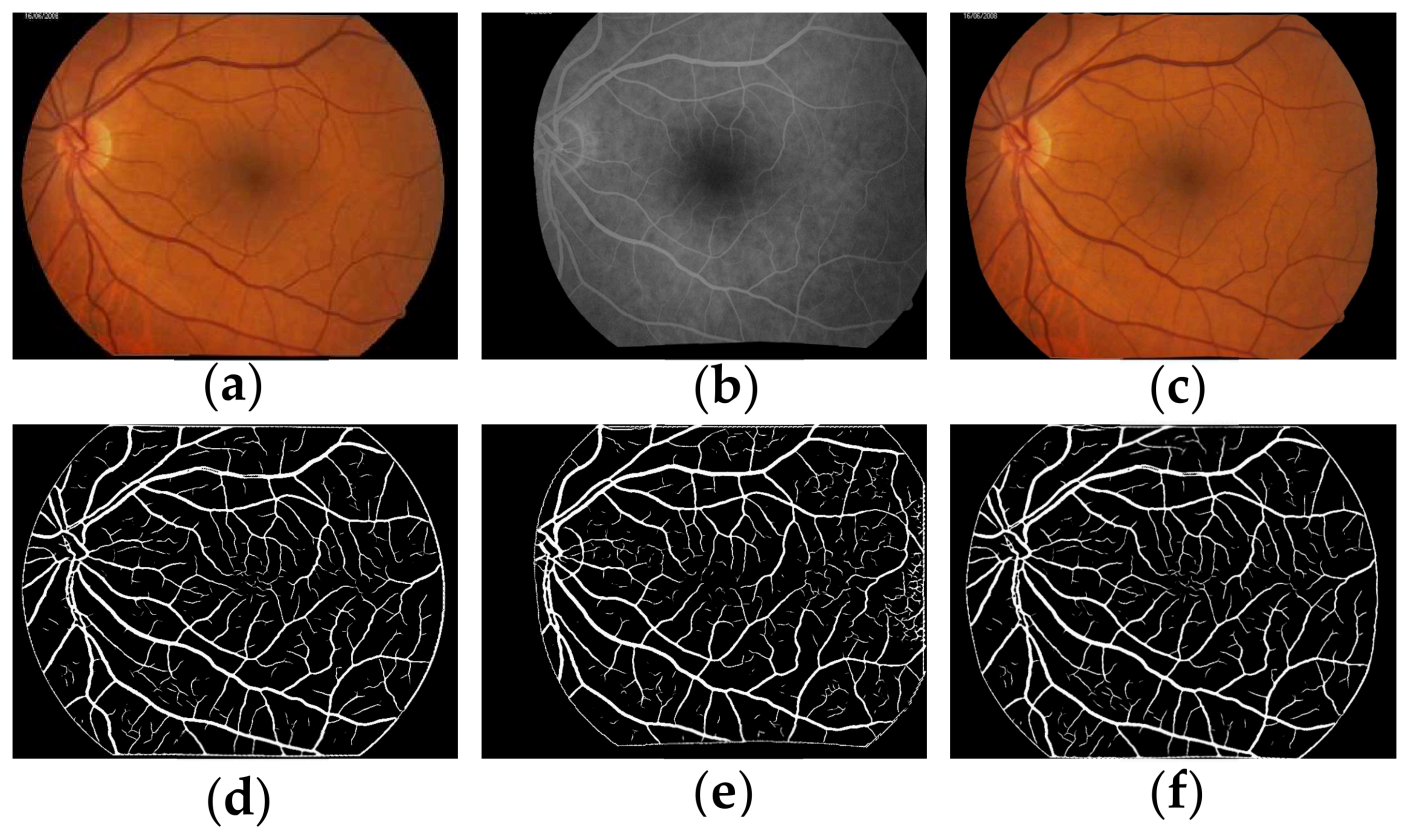
Visualization of the joint segmentation module and registration: (**a**) fixed image; (**b**) moving image; (**c**) warped moving image; (**d**) segmentation result of the fixed image; (**e**) segmentation result of the moving image; and (**f**) warped moving segmentation map.

Figure 6 shows the dense vascular segmentation mask generated by the network, as well as the warping registration results driven by the deformation field. The figure demonstrates the effectiveness of the joint segmentation module in cross-modal scenarios.

Figure 6a,b show the input CFP and FFA images, while Figure 6d,e show the corresponding vascular masks output by the joint segmentation module. A comparison reveals that this module overcomes cross-modal differences in grayscale and contrast to extract vascular features with consistent topological structures. Furthermore, Figure 6c,f show the warped moving image generated by the deformation field and its corresponding segmentation map, respectively, confirming that the semantic features extracted by this module can provide reliable guidance for subsequent spatial registration.

### 3.3. Semantically Driven Registration

Because retinal registration datasets typically lack densely annotated true deformation fields [37], this study employed a weakly supervised strategy to train a deformable registration network [48]. Corresponding vascular structures from different modalities should be spatially close but not perfectly aligned. The registration module fuses the semantic features from both modalities to capture their structural differences. Specifically, the concatenated 128-channel feature map *G* = Concat($G_{A}$, $G_{B}$) is input into the UNetFlow network. This network employs an encoder–decoder architecture, outputting a dense flow field ϕ = UnetFlow(G; $\theta_{1}$; $\theta_{2}$), where $\theta_{1}$ denotes parameters of the U-Net component and ϕ denotes the displacement vector at each pixel location. Subsequently, the moving image $F_{A}$ is deformed according to the flow field ϕ using an STN, yielding the registered image $F_{A}^{\prime }$ = STN($F_{A}$, ϕ), thereby achieving non-rigid spatial registration.

To ensure reliable registration, the training objective includes a content loss, denoted $L_{NCC}$, based on normalized cross-correlation (NCC) to measure structural similarity between the deformed image $F_{A}^{\prime }$ and the fixed image $\;F_{B}$, following the formulation in VoxelMorph [10]:

(4) $$L_{NCC}=NCC\left(F_{A}^{\prime },F_{B}\right),where\;F_{A}^{\prime }=STN(F_{A},\phi )$$

An SSIM loss on the warped image pair is additionally applied as an appearance-level auxiliary term:

(5) $$L_{ssim}=1-SSIM(F_{A}^{\prime },F_{B})$$

To suppress unreasonably abrupt changes in the flow field, a smoothing loss term can be introduced [10], adopting the diffusion-regularization-based design from VoxelMorph, as follows:

(6) $$L_{smooth}=\;\sum_{p\in \Omega }{∥\nabla \phi (p)∥}^{2}$$

Here, ∇ϕ(p) can be approximated using finite difference methods in the neighborhood to enhance the spatial continuity of the deformation field [49]. The total multi-task training objective is defined once as:

(7) $$L_{total}=\;L_{style}\left(S_{A}^{\prime },I_{style}\right)+L_{style}\left(S_{B}^{\prime },I_{style}\right)+\lambda_{1}L_{rot}+\lambda_{2}L_{NCC}\left(F_{A}^{\prime },F_{B}\right)+\lambda_{3}L_{smooth}\left(\phi \right)+\lambda_{4}L_{ssim}\left(F_{A}^{\prime },F_{B}\right)$$

where $\lambda_{1}=\lambda_{2}=1\times 10^{-3},\;\;\lambda_{4}=1\times 10^{-5}$, and $\lambda_{3}$ increased from $2\;\times 10^{-5}$ to $5\;\times 10^{-5}$ after $3\times 10^{4}$ iterations.

## 4. Experiment

### 4.1. Dataset and Preprocessing

The proposed method was evaluated on a publicly available multimodal retinal image dataset released by the Medical Image and Signal Processing Research Center, Isfahan University of Medical Sciences [50]. This dataset consists of 59 pairs of CFP and FFA images with a spatial resolution of 720 × 576 pixels: 29 pairs were acquired from 29 healthy individuals and 30 pairs from 30 patients with diabetic retinopathy, with each pair corresponding to a distinct subject; therefore, no subject appears in more than one pair or across data partitions. Among the pathological cases, lesions include microaneurysms, hemorrhages, and exudates.

Following the conventional coarse-to-fine registration routine, each image pair was first coarsely aligned using an affine transformation estimated from three manually annotated corresponding point pairs; these coarse correspondences thereby serve as the weak supervision for training the deformable registration model. All subsequent deformable registration operates on these coarsely aligned pairs.

In the experiments, the 59 image pairs were divided using two strategies. First, pairs with odd file indices (30 pairs) were used for training, while the remaining 29 pairs served as an independent test set; this split follows the protocol of prior work on this dataset [37]. Second, to reduce bias from a single data split, 5-fold cross-validation was performed on the entire dataset. All hyperparameters, loss weights, and training-schedule settings were selected on the training data only; the test set was never used for model or hyperparameter selection.

The single-channel grayscale images were duplicated to three channels to match the input requirements of the pre-trained VGG-19 network. To mitigate overfitting on the limited training set, random-deformation-based data augmentation was applied during training: coarse deformation fields were generated as 2 × 4 × 3 arrays sampled from a normal distribution (mean of 0; standard deviation of 5 pixels) and interpolated to the image resolution before being applied to each training pair.

The experiments were implemented in Python 3.7 using the PyTorch 1.7.1 framework on hardware consisting of an Intel Core i9-10940X CPU, 128 GB RAM, and an NVIDIA TITAN RTX GPU with 24 GB memory. The pre-trained VGG-19 encoder weights were frozen, and only the parameters of the joint segmentation module and registration branch were updated end-to-end. The network was optimized using the Adam optimizer with an initial learning rate of $1\times 10^{-3}$ and a batch size of 1. Each training batch consists of one CFP–FFA image pair (two images). With 30 training pairs, one epoch contains 30 iterations, and the model was trained over 6000 epochs, resulting in $1.8\times 10^{5}$ total optimization iterations. The spatial transformation parameters in the registration network were set as s=5 and t=2. The weights of the joint loss function were empirically set as $\lambda_{1}=\lambda_{2}=1\times 10^{-3}$ and $\lambda_{4}=1\times 10^{-5}$. Among them, the flow field smoothing constraint weight $\lambda_{3}$ was initially set to $2\times 10^{-5}$, and increased to $5\times 10^{-5}$ after $3\times 10^{4}$ iterations to obtain a smoother registration domain. All comparison methods were tested in the same hardware environment and default parameter settings to ensure the objective comparability of the experimental results.

### 4.2. Evaluation Metrics

Given the difficulty of obtaining ground truth deformation fields for multimodal retinal image datasets, we employed Structural Similarity Index (SSIM), the Dice coefficient, and Mean Absolute Error (MAE) to evaluate deformation registration performance in the absence of ground truth. Ablation experiments were designed for validation.

We used the SSIM metric [51] to evaluate the similarity of registered image pairs at the perceptual level, defined as follows:

(8) $${SSIM}_{\;(x,y)}=\;\frac{\left(2_{\mu_{x}\mu_{y}}+C_{1}\right)\left(2_{\sigma_{xy}}+C_{2}\right)}{\left(\mu_{x}^{2}+\mu_{y}^{2}+C_{1}\right)\left(\sigma_{x}^{2}+\sigma_{y}^{2}+C_{2}\right)}$$

where $\mu_{x}$ and $\mu_{y}$ denote the mean pixel values of images *x* and *y*, respectively; $\sigma_{x}^{2}$ and $\sigma_{y}^{2}$ denote the pixel variances of images *x* and *y*, respectively; $\sigma_{xy}$ denotes the pixel covariance between images *x* and *y*; and $C_{1}$ and $C_{2}$ denote constants used to maintain stability. The SSIM value range from −1 to 1, with higher values indicating greater similarity between two images.

The Dice coefficient was introduced as a key evaluation metric to quantify the spatial registration accuracy of the vascular networks [52]. The Dice coefficient is a widely used statistical method to measure the similarity between two sets, and could be used to assess the degree of overlap between the aligned and fixed images.

The Dice coefficient is a common metric in image segmentation and registration tasks and can be defined as follows:

(9) $$Dice=\frac{2\left|A\cap B\right|}{\left|A\right|+\left|B\right|}$$

where *A* and *B* denote the vascular regions of two images with values ranging from [0, 1]. Values closer to 1 indicate better registration quality. In this work, the vascular regions A and B were obtained with B-COSFIRE [53], a trainable but annotation-independent filter-based method, ensuring that the overlap metric is computed independently of the proposed network.

To address the limitations of the Dice coefficient in binary assessment of highly deformed regions, such as lesion occlusion in multimodal images (CFP and FFA), this paper introduces the normalized mean absolute error (MAE) to quantify pixel-level registration errors in the vascular semantic space, defined as:

(10) $$MAE=\frac{1}{N}\sum_{i=1}^{N}\left|P_{reg}\left(P_{i}\right)-P_{ref}\left(P_{i}\right)\right|$$

In this equation, N represents the total number of pixels, while $P_{reg}\left(P_{i}\right)$ and $P_{ref}\left(P_{i}\right)$ denote the normalized B-COSFIRE vesselness responses (ranging from 0 to 1) at pixel $P_{i}$ in the registered image and the fixed image, respectively. The smaller this value, the higher the absolute accuracy of the registration.

In addition, statistical significance was assessed on the per-pair metric differences over the 29 test pairs. Paired t-tests were applied when the differences were normally distributed (Shapiro–Wilk test) and Wilcoxon signed-rank tests otherwise; the resulting *p*-values were adjusted for multiple comparisons using the Holm method. Exact test statistics, raw and adjusted *p*-values, 95% confidence intervals of the mean paired difference, and effect sizes (Cohen’s dz) are provided in Supplementary Tables S1–S3.

### 4.3. Registration Results

**Figure 7:**
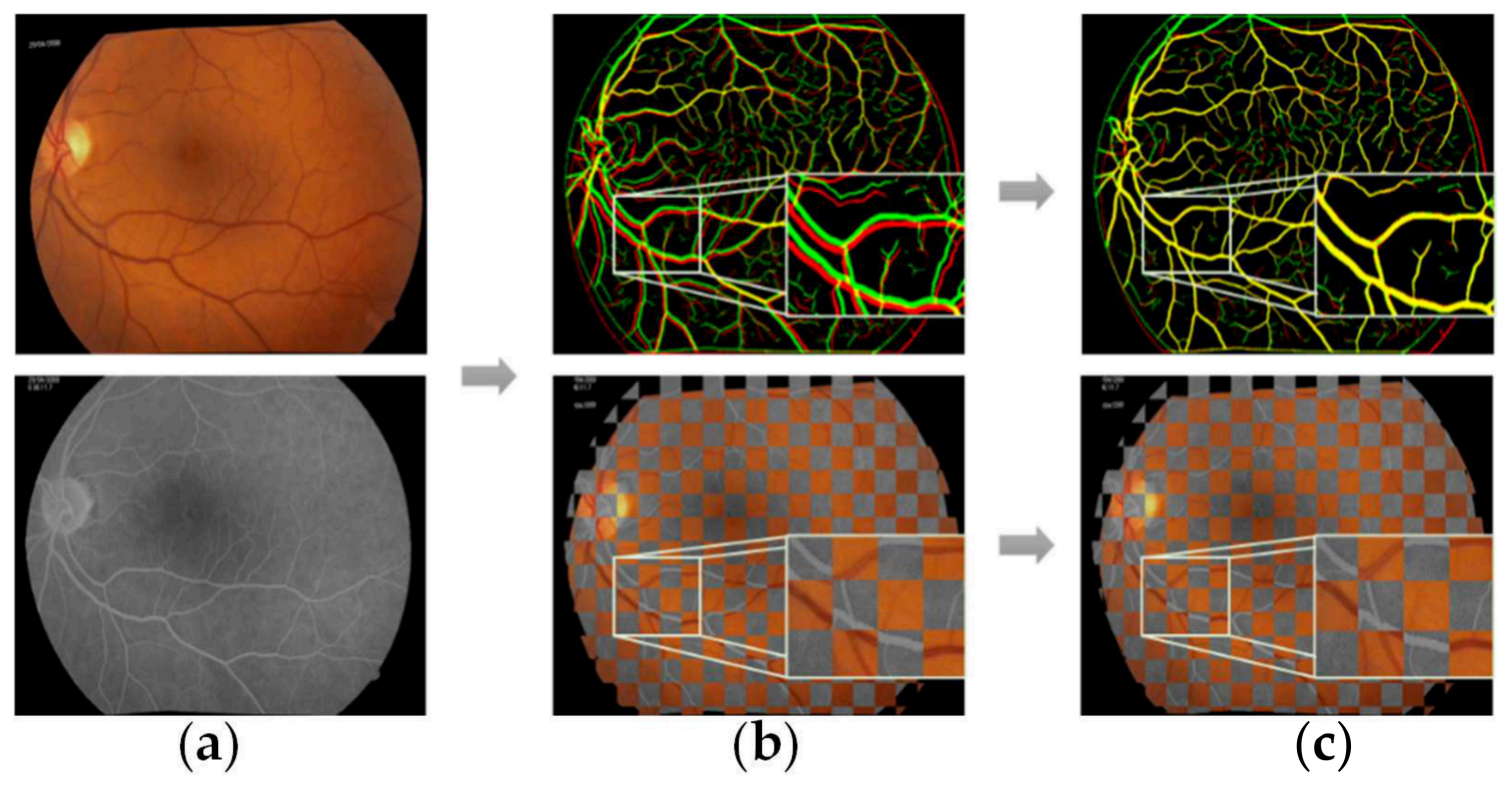
CFP and FFA images: (**a**) original images; (**b**) pre-registration; and (**c**) post-registration.

To visually demonstrate the registration results, we compared the images at the vascular and structural levels, as shown in Figure 7. Figure 7a presents the original CFP and FFA images, which exhibit considerable differences in intensity distribution and texture features. Comparison of the overlaid vascular images before and after registration (Figure 7b-top, Figure 7c-top) reveals that, despite originating from the same perspective, inherent nonlinear deformation between modalities caused substantial misregistration between the red and green vessels (CFP and FFA segmentation results) prior to registration, with sparse overlapping yellow regions. After registration, the two images were merged into a continuous yellow vascular network. Moreover, comparison of the checkerboard visualization results (Figure 7b-bottom, Figure 7c-bottom) reveals that the pre-registration images exhibited extensive misregistration and discontinuities at grid intersections; post-registration restored high continuity with smooth transitions between the vessels and background tissues.

**Figure 8:**
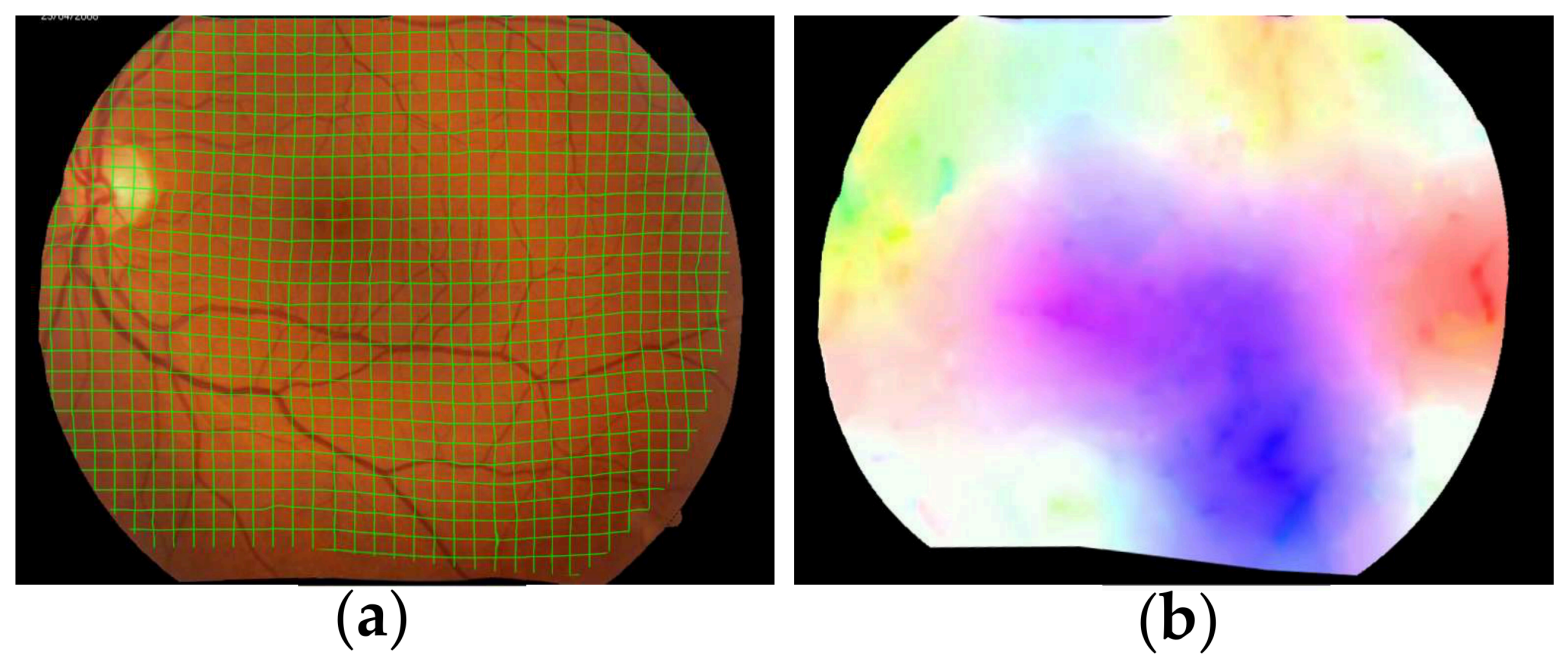
Visualization of the non-rigid deformation field predicted by the model: (**a**) deformation mesh superimposed on the reference fundus image; (**b**) pseudocolor map of the dense flow field (HSV encoding).

To verify whether the deformation field generated by the model is smooth and continuous, the deformed mesh and the dense flow field were visualized, as shown in Figure 8. Figure 8a shows the visualization results of a regular mesh superimposed on a reference fundus image after being distorted by the deformation field. Around the optic disc and in areas of dense vasculature, the mesh lines smoothly follow the tissue structure. No grid line intersections, knots, or folds were observed in the visualized deformation meshes; quantitatively, non-positive Jacobian determinants occurred in only 0.07% of pixels (~8.4 × 10^3^ out of ~1.2 × 10^7^ pixel locations over the 29 test pairs; det(J) = 0.98 ± 0.06), indicating nearly fold-free deformations. Figure 8b shows the corresponding pseudo-color map of the dense flow field, encoded in the HSV color space (where hue represents the direction of deformation, and saturation and brightness represent the intensity of deformation). The color transitions in the figure (smoothly transitioning from purple at the center to red on the right and blue at the bottom) are gentle and continuous, with no abnormal jumps or obvious noise, further verifying the spatial smoothness and continuity of the displacement field.

**Figure 9:**
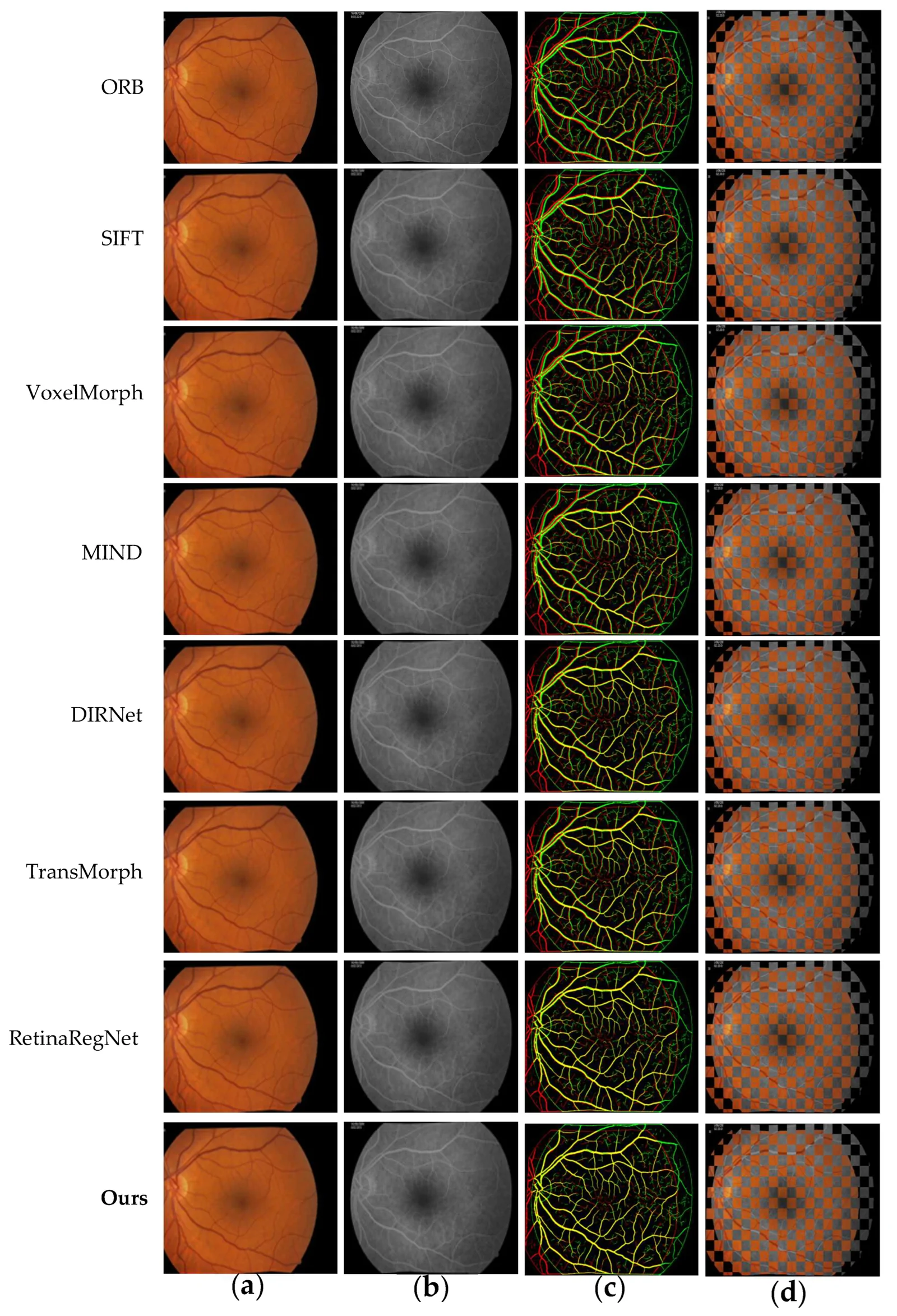
Visual comparison of registration results using different methods: (**a**) CFP image; (**b**) FFA image; (**c**) vascular overlay matching results; and (**d**) checkerboard stitching results.

Figure 9 visually demonstrates the spatial registration performance of each method on multimodal images. In the vascular superimposition shown in Figure 9c, the source and target vessels are marked in red and green, respectively, with the yellow regions indicating areas of overlap. Observation reveals that the comparison algorithm (top rows) exhibits significant red-green separation artifacts in areas of dense vasculature and fine terminal branches, with considerable local misregistration; in contrast, the images registered by our method present a large, continuous yellow vascular network with high spatial overlap. In the checkerboard visualization shown in Figure 9d, the comparison algorithms exhibit anatomical discontinuities and misregistrations at grid boundaries, whereas our method ensures smooth structural transitions in these boundary regions. This demonstrates that the deformation field generated by our model is not only stable but also achieves greater precision in local spatial registration.

These qualitative findings corroborate the quantitative evaluation metrics, validating the effectiveness and robustness of VesselRegNet’s multi-scale feature fusion process, semantically driven registration, and joint optimization strategy, which provides a solid foundation for the application of the proposed method in clinical ophthalmic diagnosis.

To evaluate the registration performance of VesselRegNet, we compared it with traditional feature extraction algorithms (ORB [54], SIFT [7]) and deep learning-based deformable registration models (VoxelMorph [10], DIRNet [55], MIND [56], TransMorph [11], RetinaRegNet [57]). The evaluation metrics on 29 test pairs are shown in Table 1.

As shown in Table 1, traditional feature matching algorithms (ORB and SIFT) are severely limited in cross-modal registration tasks, with Dice coefficients of only 0.241 and 0.306, respectively. This is due to nonlinear intensity inversion and differences in grayscale mapping between CFP and FFA, which make it easy to extract false feature points and severely limit matching performance. In contrast, deep learning models significantly improved overall performance through data-driven learning (Dice > 0.590), with RetinaRegNet—designed specifically for retinal registration—achieving the second-best results among the baselines (Dice: 0.638 ± 0.053, SSIM: 0.629 ± 0.047). The proposed VesselRegNet achieved the best registration accuracy, with Dice and SSIM reaching 0.664 ± 0.049 and 0.645 ± 0.042, respectively. Compared to the second-best RetinaRegNet, its mean values improved by 0.026 and 0.016, respectively, and the smaller standard deviation reflects the network’s higher registration stability across different samples. Paired tests confirmed that these improvements are statistically robust: our model outperforms the strongest baseline (RetinaRegNet) with Holm-adjusted *p* = 0.0039 for Dice and *p* = 0.0121 for SSIM, and every other comparison remains significant after correction (adjusted *p* < 0.001); effect sizes (Cohen’s dz) range from 0.58 to 3.58 for Dice and from 0.50 to 1.83 for SSIM (Supplementary Figure S1 and Tables S1 and S2). These results validate the effectiveness of the multi-scale semantic feature fusion mechanism in overcoming cross-modal representation differences. Landmark-based evaluation further corroborates these findings: the TRE decreased from 27.3 ± 9.2 px before registration and 12.0 ± 5.2 px after affine pre-alignment to 5.1 ± 2.5 px with our full model (Holm-adjusted *p* < 0.001 for all five comparisons; Supplementary Table S3). In terms of efficiency, ultra-lightweight methods like VoxelMorph achieved shorter inference times (0.15 s) but lower registration accuracy (Dice = 0.593). In contrast, VesselRegNet strikes a better balance between accuracy and speed: compared to the strongest baseline (RetinaRegNet), our method improves the Dice coefficient from 0.638 to 0.664 while reducing the per-pair inference time from 15.5 s to 0.42 s (an ≈37 × speedup). With an average processing time of 0.42 s per pair, the computational overhead remains well within the practical range for offline clinical analysis.

To eliminate evaluation bias caused by single-fold data partitioning and to verify the model’s generalization ability with limited samples, this paper employed 5-fold cross-validation. The quantitative results are shown in Table 2.

The results of the cross-validation experiments show that the mean Dice score was 0.659 (standard deviation 0.008) and the mean SSIM was 0.640 (standard deviation 0.006). The minimal performance fluctuations demonstrate that the model maintains stable registration accuracy across different data partitions and exhibits good generalization performance.

Real-world clinical retinal images often contain pathological features such as hemorrhages and exudates. These regions exhibit strong nonlinear deformations and are prone to structural noise interference. To evaluate the model’s robustness under extreme conditions, we selected a typical healthy sample with moderate deformation (Case A) and a highly deformed sample with extensive lesion occlusion (Case B) for independent quantitative evaluation. The results are shown in Table 3. In the healthy sample Case A, all deep learning models, except for the traditional SIFT algorithm, demonstrated a certain degree of registration capability. VoxelMorph and TransMorph achieved Dice scores of 0.612 and 0.630, respectively, while the proposed method, VesselRegNet, achieved the optimal score of 0.679, with the normalized MAE reduced to 0.069. This indicates that in routine scenarios without severe pathological interference, deep learning models can effectively handle basic deformations.

In the pathological sample Case B, the performance of all comparison methods deteriorated significantly. The VoxelMorph model, based on traditional image grayscale similarity, is susceptible to interference from lesions and plaques, leading to erroneous stretching of the deformation field; its Dice score drops to 0.563, and the normalized MAE rises to 0.110. RetinaRegNet, designed specifically for retinal registration, was also limited to a Dice score of 0.616. VesselRegNet maintained a Dice score of 0.652 even under strong deformation interference, with the lowest normalized MAE (0.078) and well-aligned vascular structures. This indicated that the front-end joint segmentation module filtered out interference from pathological tissue, maintaining the spatial stability of the deformation field in complex regions.

**Figure 10:**
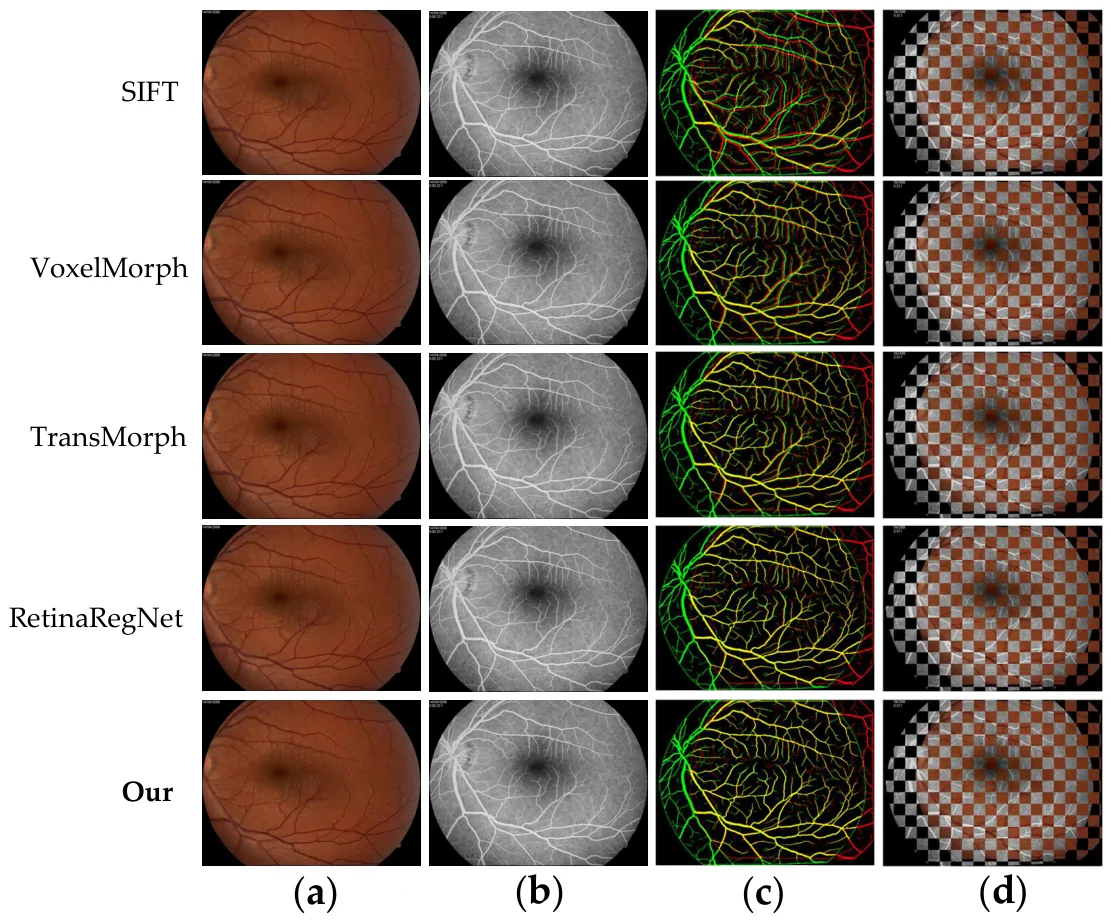
Visual comparison of registration results for Case A: (**a**) CFP image; (**b**) FFA image; (**c**) vascular overlay matching results; and (**d**) checkerboard-style stitching results.

As shown in Figure 10, a visual comparison of the registration performance of various algorithms on the Case A sample is presented. The fundus structure in this sample is relatively healthy, and the distortion primarily stems from differences in the shooting angles. Observation reveals that the traditional SIFT algorithm, due to its difficulty in handling cross-modal imaging differences, exhibits extensive separation of red and green blood vessels and grid misalignment; in contrast, most deep learning models demonstrate a certain degree of alignment capability. The VesselRegNet proposed in this paper produces the most continuous and dense yellow overlay network in the vascular superimposition shown in Figure 10c, and exhibits the smoothest edge transitions in the checkerboard-style stitching shown in Figure 10d. This demonstrates that the model achieves optimal anatomical alignment accuracy in baseline scenarios without severe pathological interference.

**Figure 11:**
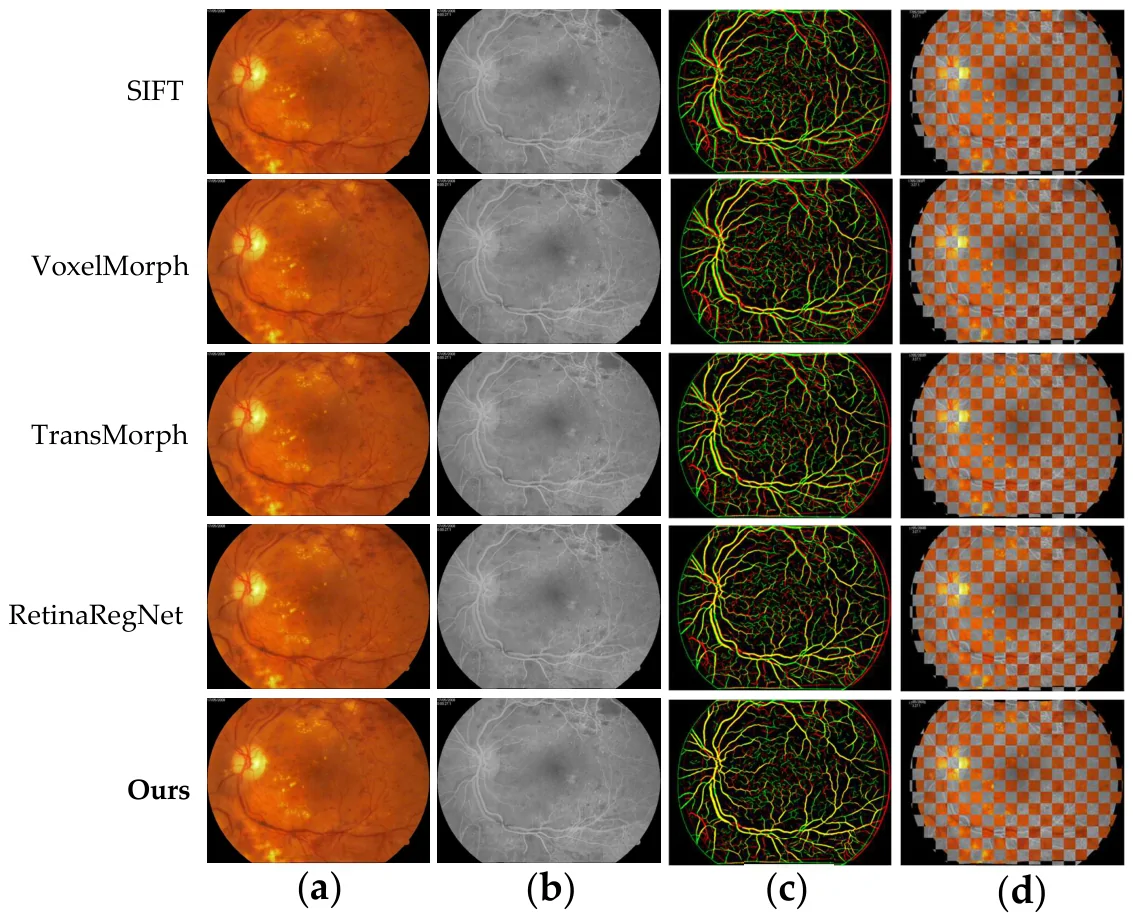
Visual comparison of registration results for Case B: (**a**) CFP image; (**b**) FFA image; (**c**) vascular overlay matching results; and (**d**) checkerboard-style stitching results.

Figure 11 focuses on a highly pathological Case B sample with extensive lesion occlusion, demonstrating the models’ robustness to interference under extreme conditions. Observation reveals that the distinct bright yellow pathological lesions on the left side of the CFP image in Figure 11a exhibit a starkly different grayscale appearance in the FFA image of Figure 11b. This significant cross-modal discrepancy directly causes models like VoxelMorph, which rely on global grayscale information, to exhibit vascular ghosting and erroneous stretching around the lesion. Even RetinaRegNet, which is optimized for retinal features, exhibits localized red-green separation and loss of alignment accuracy at the edges of dense lesions. In contrast, our method uses vascular semantic features extracted in the preprocessing stage as precise guidance, effectively mitigating the misleading grayscale patterns of pathological tissue. As shown in Figure 11c,d, the vessels in dense lesion areas still exhibit a high degree of yellow overlap, and no abnormal tearing of the checkerboard pattern is observed, demonstrating the robustness of this framework in complex real-world clinical scenarios.

### 4.4. Ablation Experiments

To investigate the impact of different mechanisms on registration performance, this section conducted incremental and combinatorial ablation experiments on the test set; the quantitative results are shown in Table 4.

After removing the encoder skip connection (Remove Skip), the Dice score decreased by 5.3%, while the SSIM score decreased by only 1.0%. This indicates that the loss of local spatial details caused by network downsampling primarily affects the registration accuracy of microvessels. When the flow field prediction mechanisms were weakened (Reduced Flow and Single Scale), the decrease in Dice was minor (−0.1% and −2.0%), but SSIM showed a significant decline (−5.5% and −10.1%). From the perspective of physical deformation, when multi-scale high-frequency flow field constraints are absent, the model forces registration of the main vascular trunks, causing excessive stretching of the surrounding parenchymal tissue and resulting in compromised global structural fidelity. Combined ablation experiments showed that when both skip connections and the multi-scale mechanism were removed simultaneously (Remove Skip + Single Scale), registration performance deteriorated most severely (Dice decreased by 8.4%, SSIM decreased by 13.0%). These results indicate that skip connections (responsible for preserving vascular details) and the multi-scale flow field (responsible for controlling smooth deformation) are both indispensable. Only by combining these two components can the model accurately align blood vessels while avoiding unreasonable physical distortion of retinal tissue.

## 5. Conclusions

Multimodal retinal image registration is important in medical image analysis. To address the limitations of traditional methods in feature representation and the restricted robustness of deep-learning methods to multimodal differences and complex deformations, this study proposed an end-to-end joint segmentation and registration network called VesselRegNet. The experimental results demonstrated that VesselRegNet achieved optimal performance on a test dataset comprising healthy and pathological samples with an average Dice coefficient of 0.664 and an SSIM of 0.645, notably outperforming traditional landmark-based methods and current mainstream deep-learning registration models. Ablation studies further validated the effectiveness of key design elements, including multi-scale fusion, skip connections, and semantic feature-driven processing.

Several limitations should be noted. First, the dataset contains 59 pairs from a single center, and all pathological cases represent diabetic retinopathy; generalization to other pathologies requires further multi-center validation. Second, the pipeline relies on coarse affine pre-alignment based on three manually annotated point pairs; fully automatic coarse alignment is left to future work. Third, overlap-based metrics computed on vessel maps cannot fully verify geometric fidelity; we therefore additionally report landmark-based target registration error as an independent geometric measure (Section 4.3 and Supplementary Table S3). Finally, the dataset provides no pixel-wise vessel annotations; our segmentation is supervised only through style transfer from an external target, which may bound achievable segmentation fidelity.

In summary, this study demonstrated the considerable potential of multi-scale feature fusion and joint learning strategies for enhancing the accuracy of multimodal retinal image registration.

## Figures and Tables

**Table 1:** Comparative registration results of different methods.

Methods	Dice	SSIM	Params (M)	FLOPs (G)	Time/Pair (s)
ORB [54]	0.241 ± 0.105	0.539 ± 0.073	N/A ^1^	N/A ^1^	0.45 ± 0.12
SIFT [7]	0.306 ± 0.098	0.552 ± 0.069	N/A ^1^	N/A ^1^	0.68 ± 0.18
VoxelMorph ^2^ [10]	0.593 ± 0.062	0.577 ± 0.056	1.2	4.0	0.15 ± 0.04
MIND [56]	0.602 ± 0.059	0.599 ± 0.052	N/A ^1^	N/A ^1^	0.85 ± 0.25
DIRNet [55]	0.604 ± 0.058	0.601 ± 0.051	1.6	4.5	0.18 ± 0.05
TransMorph [11]	0.622 ± 0.056	0.618 ± 0.048	15.0	20.0	0.30 ± 0.08
RetinaRegNet ^3^ [57]	0.638 ± 0.053	0.629 ± 0.047	—	—	15.5 ± 2.5
**Ours**	**0.664 ± 0.049**	**0.645 ± 0.042**	6.2 ^a^	24.0 ^b^	0.42 ± 0.09

^1^ Non-learning methods without trainable parameters. ^2^ The original unsupervised CNN architecture of VoxelMorph, adapted to 2D inputs. ^3^ RetinaRegNet is a zero-shot method requiring no task-specific training; its inference cost is dominated by pretrained feature extraction. ^a^ Trainable parameters only (multi-scale fusion decoder, shared prediction head, and registration network); the frozen VGG-19 encoder contributes ≈ 20 M additional parameters, giving ≈ 26.2 M in total. ^b^ FLOPs of the trainable modules only; the full forward pass including the frozen VGG-19 encoder requires ≈ 310 G. All methods were evaluated on the same workstation (Intel Core i9-10940X, NVIDIA TITAN RTX 24 GB); runtime is averaged over the 29 test pairs including deformation-field prediction and warping. Values are presented as means ± standard deviations over the 29 test pairs. N/A: not applicable.

**Table 2:** Five-fold cross-validation results for the VesselRegNet model.

Fold	Sample Size	Dice	SSIM
Fold-1	12	0.649	0.632
Fold-2	12	0.662	0.641
Fold-3	12	0.655	0.636
Fold-4	11	0.669	0.649
Fold-5	12	0.660	0.641
Mean ± Std	—	0.659 ± 0.008	0.640 ± 0.006

**Table 3:** Comparison of registration performance for the example case under different degrees of deformation.

Case Type	Methods	Dice	SSIM	Normalization MAE
Case A	SIFT	0.332	0.565	0.139
	VoxelMorph	0.612	0.584	0.094
	TransMorph	0.630	0.622	0.083
	RetinaRegNet	0.646	0.632	0.077
	VesselRegNet	0.679	0.653	0.069
Case B	SIFT	0.268	0.541	0.163
	VoxelMorph	0.563	0.551	0.110
	TransMorph	0.593	0.604	0.095
	RetinaRegNet	0.616	0.619	0.089
	VesselRegNet	0.652	0.636	0.078

SSIM: structural similarity index; MAE: mean absolute error.

**Table 4:** Ablation experiment results.

Experimental Setup	Dice	SSIM	Dice (%)	SSIM (%)
Baseline	0.664 ± 0.049	0.645 ± 0.042	—	—
Remove Skip	0.629 ± 0.055	0.639 ± 0.044	−5.3	−1.0
Reduced Flow	0.663 ± 0.050	0.610 ± 0.047	−0.1	−5.5
Single Scale	0.650 ± 0.053	0.580 ± 0.049	−2.0	−10.1
Reduced Flow + Single Scale	0.633 ± 0.057	0.571 ± 0.052	−4.6	−11.5
Remove Skip + Reduced Flow	0.620 ± 0.059	0.597 ± 0.052	−6.5	−7.5
Remove Skip + Single Scale	0.608 ± 0.061	0.562 ± 0.054	−8.4	−13.0
Binary-Mask Guidance	0.617 ± 0.047	0.591 ± 0.043	−7.1	−8.4
Remove Rotation Loss	0.642 ± 0.043	0.618 ± 0.039	−3.3	−4.2

SSIM: structural similarity index.

## Data Availability

The data presented in this study are openly available as a multimodal retinal image at https://misp.mui.ac.ir/fa/node/1399 (accessed on 23 October 2025), reference [50].

## Supplementary Materials

The following supporting information can be downloaded at: https://www.mdpi.com/article/10.3390/bioengineering13101179/s1, Table S1: Paired statistical analysis of Dice scores over the 29 test pairs; Table S2: Paired statistical analysis of SSIM scores over the 29 test pairs; Table S3: Landmark-based target registration error (TRE) over the 29 test pairs; Figure S1: Paired differences in Dice and SSIM between our model and each baseline over the 29 test pairs.

## Abbreviations

The following abbreviations are used in this manuscript:

BCE	Binary Cross-Entropy
CC	Cross-Correlation
CFP	Color Fundus Photography
CNN	Convolutional Neural Network
DRIU	Deep Retinal Image Understanding
DR	Diabetic Retinopathy
FFA	Fluorescein Angiography
HSV	Hue, Saturation, Value
ICP	Iterative Closest Point
MAE	Mean Absolute Error
MI	Mutual Information
MIND	Modality Independent Neighbourhood Descriptor
NCC	Normalized Cross-Correlation
OCTA	Optical Coherence Tomography Angiography
OCT	Optical Coherence Tomography
ORB	Oriented FAST and Rotated BRIEF
RVO	Retinal Vein Occlusion
SIFT	Scale-Invariant Feature Transform
SSIM	Structural Similarity Index
STN	Spatial Transformer Network
SURF	Speeded-Up Robust Features
