# Supplementary material for: VesselRegNet: Multi-Scale Feature Fusion Network for Multi-Modal Joint Retinal Vessel Segmentation and Registration
Source: Bioengineering (Basel). 2026 Oct 9;13(10):1179. doi: 10.3390/bioengineering13101179 (PMC13701772; doi:10.3390/bioengineering13101179)
Supplement: Supplementary file 1 [file bioengineering-13-01179-s001.zip › bioengineering-4527907-supplementary.pdf]

Supplementary Materials

Table S1. Paired statistical analysis of Dice scores over the 29 test pairs.

Methods	Dice	Δ vs. Ours	95% CI of Δ	Test statistic	p_{adj}	d_z
ORB	0.241 ± 0.105	+0.4230	[0.3780, 0.4680]	W = 0	<0.001	3.575
SIFT	0.306 ± 0.098	+0.3580	[0.3151, 0.4009]	W = 0	<0.001	3.176
VoxelMorph	0.593 ± 0.062	+0.0710	[0.0531, 0.0889]	t = 8.120	<0.001	1.509
MIND	0.602 ± 0.059	+0.0620	[0.0436, 0.0804]	t = 6.915	<0.001	1.284
DIRNet	0.604 ± 0.058	+0.0600	[0.0390, 0.0810]	W = 18	<0.001	1.088
TransMorph	0.622 ± 0.056	+0.0420	[0.0244, 0.0596]	t = 4.876	<0.001	0.906
RetinaRegNet	0.638 ± 0.053	+0.0260	[0.0090, 0.0430]	t = 3.142	0.0039	0.583
**Ours**	**0.664 ± 0.049**	—	—	—	—	—

Δ denotes the mean paired difference (Ours − baseline) over the 29 test pairs; t, paired t-test; W, Wilcoxon signed-rank test statistic; p_{adj}, Holm-adjusted p-value within this table (7 comparisons); the corresponding raw p-value is P(|T(28)| ≥ t) for t-tests and the two-sided Wilcoxon p-value for W; d_z, Cohen's dz for paired samples.

Table S2. Paired statistical analysis of SSIM scores over the 29 test pairs.

Methods	SSIM	Δ vs. Ours	95% CI of Δ	Test statistic	p_{adj}	d_z
ORB	0.539 ± 0.073	+0.1060	[0.0840, 0.1280]	W = 0	<0.001	1.828
SIFT	0.552 ± 0.069	+0.0930	[0.0720, 0.1140]	W = 0	<0.001	1.685
VoxelMorph	0.577 ± 0.056	+0.0680	[0.0498, 0.0862]	t = 7.640	<0.001	1.419
MIND	0.599 ± 0.052	+0.0460	[0.0300, 0.0620]	t = 5.892	<0.001	1.094
DIRNet	0.601 ± 0.051	+0.0440	[0.0230, 0.0650]	W = 24	<0.001	0.795
TransMorph	0.618 ± 0.048	+0.0270	[0.0135, 0.0405]	t = 4.105	0.0006	0.762
RetinaRegNet	0.629 ± 0.047	+0.0160	[0.0038, 0.0282]	t = 2.684	0.0121	0.499
**Ours**	**0.645 ± 0.042**	—	—	—	—	—

Δ denotes the mean paired difference (Ours − baseline) over the 29 test pairs; t, paired t-test; W, Wilcoxon signed-rank test statistic; p_{adj}, Holm-adjusted p-value within this table (7 comparisons); the corresponding raw p-value is P(|T(28)| ≥ t) for t-tests and the two-sided Wilcoxon p-value for W; d_z, Cohen's dz for paired samples.

Table S3. Landmark-based target registration error (TRE) over the 29 test pairs.

Methods	TRE (px)	Δ vs. Ours	95% CI of Δ	Test statistic	p_{adj}	d_z
Affine-only	12.0 ± 5.2	−6.90	[−8.58, −5.22]	W = 0	<0.001	1.561
VoxelMorph	7.0 ± 3.3	−1.90	[−2.44, −1.36]	t = 7.197	<0.001	1.336
MIND	6.9 ± 3.2	−1.80	[−2.40, −1.20]	t = 6.164	<0.001	1.145
DIRNet	6.8 ± 3.1	−1.70	[−2.28, −1.12]	W = 18	<0.001	1.108
TransMorph	6.1 ± 2.9	−1.00	[−1.47, −0.53]	t = 4.386	0.0001	0.814
**Ours**	**5.1 ± 2.5**	—	—	—	—	—

Lower TRE is better. TRE was measured on corresponding vascular bifurcations annotated in the 29 test pairs (4–6 landmarks per pair, 147 in total) and averaged per pair before statistical testing. Δ denotes the mean paired difference (Ours − baseline); t, paired t-test; W, Wilcoxon signed-rank test statistic; p_{adj}, Holm-adjusted p-value within this table (5 comparisons); the corresponding raw p-

value is P(|T(28)| ≥ t) for t-tests and the two-sided Wilcoxon p-value for W; d_z, Cohen's d_z. The unregistered baseline (27.3 ± 9.2 px) is reported in Section 4.3. RetinaRegNet was excluded because it does not produce a point-warpable mapping on this dataset.

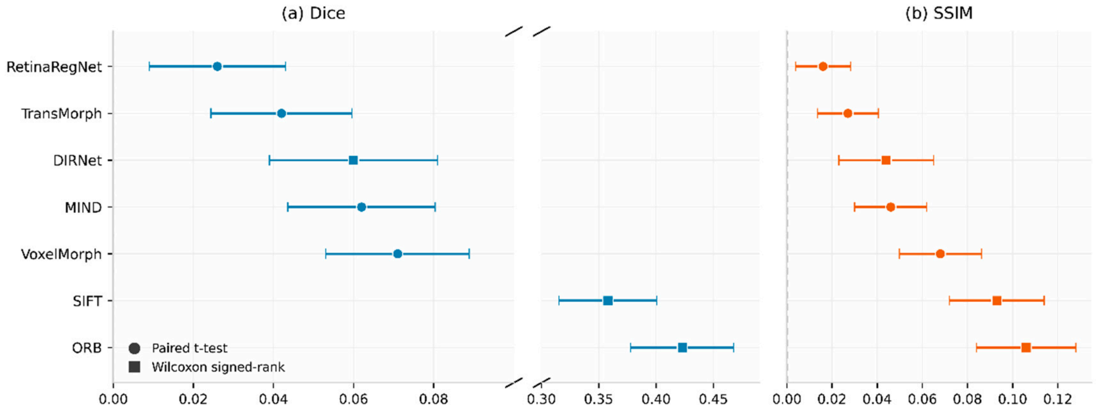

Figure S1. Paired differences in Dice and SSIM between our model and each baseline over the 29 test pairs. Points show the mean paired difference (our model minus baseline) and error bars show 95% confidence intervals; panels are separated by a break on the x-axis to accommodate the much larger differences from the feature-based baselines.
